# Evidence-based nursing interventions in cognitive dysfunction among adults with brain injury: a quasi-experiment

**DOI:** 10.7189/jogh.15.04253

**Published:** 2025-08-29

**Authors:** Yitian Gao, Wanqiong Zhou, Ying Wang, Lanshu Zhou

**Affiliations:** 1 College of Nursing, Shanghai University of Traditional Chinese Medicine, Shanghai, China; 2 Nursing Department, Shuguang Hospital affiliated with Shanghai University of Traditional Chinese Medicine, Shanghai, China; 3 Nursing Department, The First Rehabilitation Hospital of Shanghai, Shanghai, China; 4 Key Laboratory of Geriatric Long-term Care, Naval Medical University, Shanghai, China

## Abstract

**Background:**

While evidence-based interventions are increasingly used in cognitive care, their effectiveness for patients with brain injury remains not well established. Therefore, we aimed to compare the effects of an evidence-based nursing intervention programme *vs.* conventional nursing care in patients with cognitive dysfunction following brain injury.

**Methods:**

We enrolled brain injury patients from two neurosurgical wards (A and B) of a Shanghai rehabilitation hospital (July 2023–January 2024) in a non-randomised controlled trial. Ward A implemented the evidence-based cognitive intervention programme, while ward B maintained routine care. The segregated ward design prevented cross-contamination between groups. Outcomes were measured at baseline, immediately after a two-week intervention (T1), and at a four-week follow-up (T2). We analysed primary (*i.e.* the Mini-Mental State Examination (MMSE)) and secondary (*i.e.* activities of daily living (ADL) and cognitive knowledge scores) outcomes using generalised estimating equations (GEE) with terms for time, group, and their interaction. We set statistical significance at *P* < 0.05.

**Results:**

We enrolled 124 brain injury patients with cognitive dysfunction, with 117 completing the study (intervention n = 59; control n = 58; attrition = 5.6%). No significant differences were found between the groups at baseline. The GEE results showed significantly higher cognitive function improvements in the intervention group than the control group at both T1 (b = 1.71; 95% confidence interval (CI) = 0.76, 2.66; *P* < 0.001) and T2 (b = 2.71; 95% CI = 1.42, 4.00; *P* = 0.001). Results also showed a significantly greater increase in ADL (b = 4.93; 95% CI = 2.14, 7.72; *P* = 0.001) and caregivers’ cognitive scores (b = 8.09; 95% CI = 2.78, 13.41; *P* = 0.003) at T1, with sustained increases at T2 in ADL (b = 5.46; 95% CI = 2.15, 8.77; *P* = 0.001) and caregivers’ cognitive scores (b = 13.51; 95% CI = 6.76, 20.27; *P* < 0.001).

**Conclusions:**

The programme was effective in improving cognitive function and shows potential for clinical implementation and generalisation. A longer follow-up study is needed to evaluate its long-term effects on clinical outcomes.

**Registration:**

We prospectively registered this study with the Chinese Clinical Trial Registry (Registration ID: ChiCTR2300072283).

Brain injury, a prevalent neurosurgical emergency, is characterised by high morbidity, disability, and mortality rates, ranking among the leading global causes of death and disability [1,2]. Annually, brain injury affects over 50 million people worldwide, resulting in more than ten million hospitalisations/deaths and USD 400 billion in economic losses [3,4]. The USA records more than two million brain injury-related medical consultations and 64 000 deaths annually [3]. In China, incidence rates have risen to 55–64 per 100 000 population, causing approximately 100 000 deaths and hundreds of thousands of disabilities yearly [5]. China's brain injury burden exceeds that of most nations, placing significant healthcare and socioeconomic burdens on individuals, families, and society.

Cognitive dysfunction is the most prevalent and persistent complication after brain injury. The incidence of cognitive impairment reaches 40–60% in mild brain injury cases, escalating to 90% in moderate-to-severe cases [6,7]. Clinical manifestations include delayed responsiveness, altered consciousness, memory deficits, impaired auditory comprehension, spatial disorientation, agnosia, apraxia, and reduced initiative [8]. These cognitive sequelae often persist for years post-injury, representing the primary contributor to self-care limitations, disorientation, and injury recurrence in survivors [9]. Such impairments severely affect patients' physical and mental health, reduce their quality of life, and place considerable care burdens on both families and society [10]. Although contemporary brain injury treatments and rehabilitation techniques have advanced, Potvin and colleagues’ longitudinal study reveals that only about 50% of patients achieve functional cognitive improvement [11]. This underscores cognitive rehabilitation as a critical priority in neurological care.

The INCOG 2.0 Guidelines and the Chinese Expert Consensus on Neurosurgical Critical Care emphasise nurses' pivotal role as multidisciplinary team members in implementing early cognitive rehabilitation for brain injury patients [12,13]. Despite growing recognition of the importance of early cognitive rehabilitation, its clinical implementation is hindered by heterogeneous approaches (*e.g.* exercise once *vs.* twice daily), inconsistent assessment tools (*i.e.* Mini-Mental State Examination (MMSE), Montreal Cognitive Assessment, Loewenstein Occupational Therapy Cognitive Assessment), and variable intervention quality, which together impede the development of standardised nursing strategies [14,15]. Research suggests major gaps in the clinical practice of cognitive rehabilitation nursing, with many effective interventions still underused in practice and nursing’s key role in early cognitive function recovery not yet fully realised [16].

We applied the ‘Knowledge To Action’ model as a theoretical framework [17] to develop a clinically appropriate, practical, evidence-based nursing programme for early cognitive function rehabilitation in brain injury patients, based on the best available [18]. We aim to evaluate the feasibility and effectiveness of the evidence-based nursing programme in clinical practice, providing reliable evidence for clinical cognitive nursing practice, and to improve the quality of clinical care.

## METHODS

### Study design

We conducted a non-randomised controlled trial with brain injury patients from two neurosurgical wards (A and B) at a Shanghai rehabilitation hospital between July 2023 and January 2024. We assigned ward A to implement the evidence-based cognitive rehabilitation program (Table S1 in the **Online Supplementary Document**), while Ward B maintained routine care comprising admission/discharge education, medication administration, skin care, catheter management, and activity monitoring. Wards A and B are both neurosurgical wards admitting patients with brain injuries and are highly homogeneous in hardware equipment, bed capacity, and staff-to-patient ratios. The wards operated independently on separate floors (2nd *vs.* 5th) with no staff or caregiver crossover between groups. We adhered to the Standards for Quality Improvement Reporting Excellence 2.0 guidelines (Checklist S1 in the **Online Supplementary Document**) [19].

### Intervention team composition

We established a 12-member implementation team, comprising one evidence-based nursing specialist (for project framework development and methodology oversight), one nursing department director (for programme administration and operational supervision), two chief nurses (for workflow coordination, implementation process monitoring, and clinical guidance), seven staff nurses (for intervention delivery and outcome indicator review, did not participate in the formulation of intervention programmes), and one postgraduate researcher (for conducting evidence synthesis, data collection and analysis, implementation process monitoring, and protocol adjustments). The team lead (LS) provided all team members with standardised training, including patient screening protocols, assessment scale administration, intervention methods, and precautions, before implementation. All members were required to pass the instructors' (LS) competency evaluations, demonstrating proficiency in simulated patient screening and intervention scenarios before beginning clinical work.

### Study subjects

**Figure 1:**
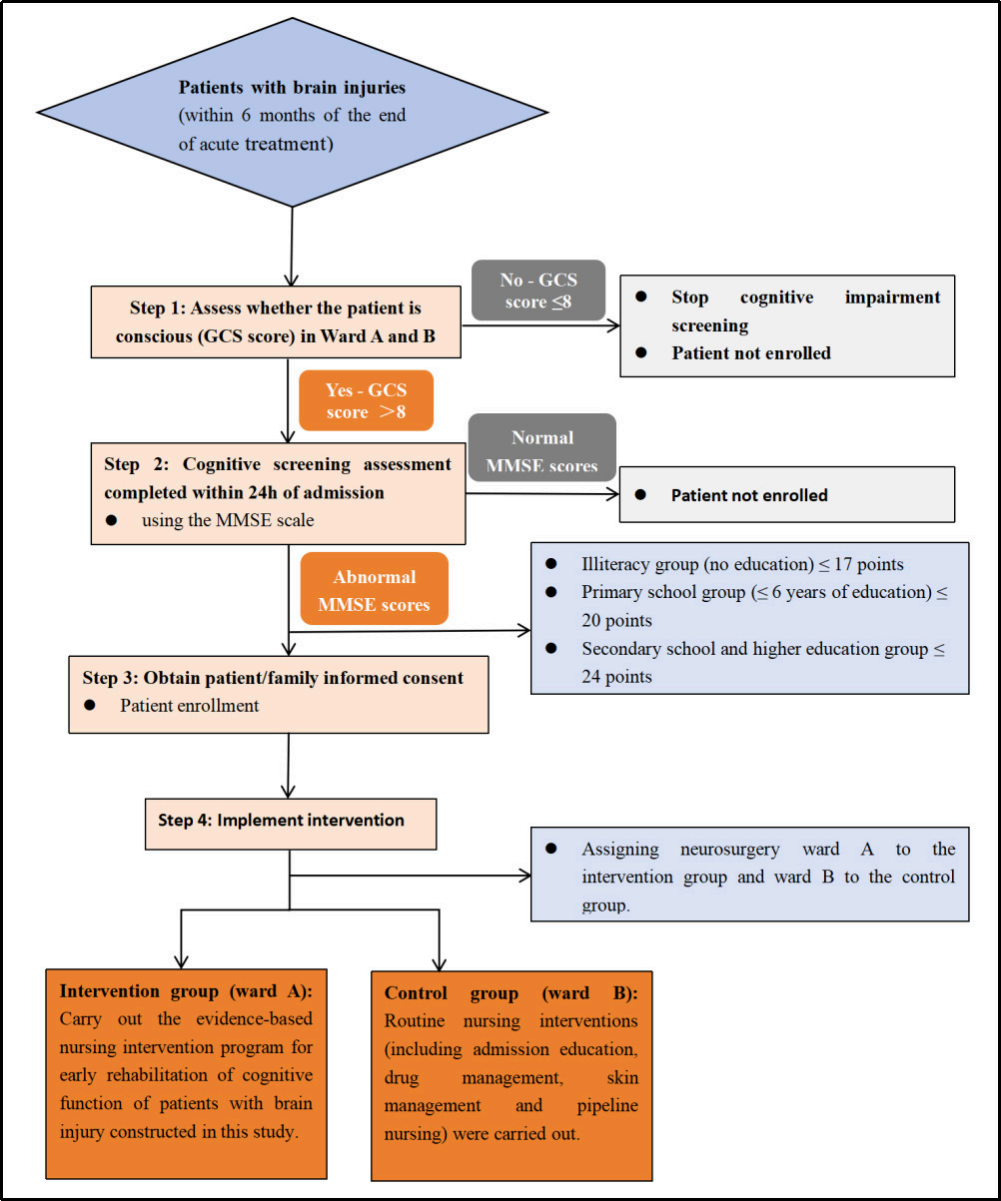
Patient enrolment screening flowchart.

Neurological function following central nervous system injury demonstrates measurable reorganisation capacity, with early rehabilitation enhancing neural plasticity and functional adaptation [20]. International evidence identifies the first six months post-brain injury as the critical rehabilitation window [20,21]. Given this neuroplastic potential, we enrolled brain injury patients within six months post-acute treatment to optimise rehabilitation outcomes (**Figure 1**) [21,22].

The inclusion criteria were: cognitive dysfunction confirmed via MMSE screening, brain injury, acute phase completion within the preceding six months with stable vital signs, age ≥18 years, and a legal guardian/provider who provided informed consent. The exclusion criteria were: Glasgow Coma Scale score of ≤8 and severe cardiopulmonary/hepatic dysfunction or active malignancies.

Using cognitive function score changes (*i.e.* MMSE) as primary outcomes, we determined the sample size via the two independent means formula [23]:



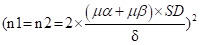



We set the significance level (α) at 0.05, representing a 5% risk of type I error, and type II error rate (β) at 0.10, corresponding to a 90% statistical power. From standard normal distribution tables, the critical values were μα = 1.96 for α and μβ = 1.282 for β. We calculated the effect size (δ = 1.94) based on the mean (x̄) difference reported by He and colleagues [24] (intervention group x̄ = 28.2; standard deviation (SD) = 2.6 *vs.* control group x̄ = 26.2; SD = 2.8), with a pooled SD of 2.78. This effect size indicated a required sample of 43 participants per group. Allowing for an anticipated 20% attrition, we aimed to recruit 54 participants per group, for a total of 108 patients.

### Measurements

We measured MMSE (primary outcome) and activities of daily living (ADL) with cognitive knowledge scores for nurses and patients' families (secondary outcomes) at baseline (T0), immediately after a two-week intervention (T1), and at a four-week follow-up (T2).

#### MMSE scale

The MMSE, developed by Folstein and colleagues [25] and cross-culturally adapted into Chinese by Wang and colleagues [26], remains the most widely implemented cognitive screening tool globally. This 30-item scale assesses five domains: orientation, attention/calculation, memory, recall, and language. Total scores range from 0 to 30, with higher scores indicating better cognitive function (Cronbach's α = 0.81). The normal cut-off value is education-stratified: >17 for illiteracy, >20 for elementary education, and >24 for secondary/higher education.

#### Modified Barthel Index Scale

The Modified Barthel Index scale, adapted by Li and colleagues from the original Barthel Index, is the tool for assessing ADL [27]. This ten-item scale assigns 0–15 points per item based on assistance requirements, with total scores ranging from 0 to 100. Higher scores indicate greater functional independence (Cronbach's α = 0.929). Dependence levels are defined as: ≤40 (severe, full assistance), 41–60 (moderate, substantial assistance), 61–99 (mild, minimal assistance), and 100 (independent).

#### Brain Injury Cognitive Rehabilitation Nursing Knowledge Questionnaire (Nurses' version)

To assess clinical nurses' cognitive intervention knowledge, the research team developed the Brain Injury Cognitive Rehabilitation Nursing Knowledge Questionnaire (Nurses' Version) (File S1 in the **Online Supplementary Document**). We evaluated content validity through an expert panel meeting with 10 specialists (Table S2 in the **Online Supplementary Document**). All experts actively participated (100% response rate), demonstrating strong engagement. The questionnaire demonstrated high expert credibility, with an authority coefficient (Cr) of 0.91 – exceeding the 0.7 threshold – calculated as the average of scoring rationale (Ca = 0.94) and content familiarity (Cs = 0.88) (Table S3 in the **Online Supplementary Document**). It also showed good content validity, with item-level content validity indices ranging from 0.8 to 1.0 and scale-level content validity index of 0.87.

The 100-point questionnaire comprises two sections: demographics (*i.e.* gender, age, professional title, and clinical experience) and knowledge assessment (*i.e.* ten multiple-choice items (ten points each) evaluating cognitive function assessment and intervention competencies). Correct answers receive full credit and incorrect responses score 0.

#### Brain Injury Cognitive Rehabilitation Knowledge Questionnaire (Patient's family version)

Given that cognitive impairment limits patients' capacity to comprehend questionnaire items and make informed choices, and considering that family caregivers serve as primary caregivers for this population, the intervention's effectiveness was also assessed through a cognitive knowledge questionnaire administered to patients' family members.

The Brain Injury Cognitive Rehabilitation Knowledge Questionnaire, based on an existing programme (Patient's Family Version) (File S2 in the **Online Supplementary Document**), assessed primary family caregivers' cognitive rehabilitation knowledge. Following the same validation protocol as previously described, the questionnaire demonstrated excellent content validity (scale-level content validity index = 0.89).

This 100-point instrument comprised two sections: caregiver demographics (*i.e.* gender, age, and relationship to patient) and knowledge assessment (*i.e.* ten true/false items (ten points each) evaluating core rehabilitation concepts). Correct answers received full credit, and incorrect responses scored 0.

### Data collection and quality control

Prior to intervention initiation, research nurses conducted comprehensive briefings for patients and families regarding study objectives and procedures. Baseline data collection commenced after obtaining written informed consent, with follow-up assessments conducted at two- and four-week intervals post-intervention. The team explicitly informed all participants of their rights to voluntary participation and unrestricted withdrawal.

### Data analysis

Two researchers (YG and WZ) independently reviewed and analysed the collected data using SPSS, Version 26.0 (SPSS Inc., Chicago, Illinois, USA) and *R*, Version 4.3.2 (RCoreTeam, Vienna, Austria). With sample sizes exceeding 50 in both intervention and control groups, the researcher employed Kolmogorov-Smirnov test to assess data normality. They used descriptive statistics for the baseline data of patients in the two groups: describing count data as frequency (%), and continuous measures that met the normal distribution as x̄ (SD), and those not meeting the normal distribution as median (interquartile range). For intergroup comparisons of baseline data, they analysed categorical variables using χ^2^ tests, and continuous variables with independent samples *t* tests when normally distributed and Mann-Whitney U tests when normality assumptions were violated.

When comparing indicators across different time points, the researchers used generalised estimating equations (GEE) model to analyse data from both groups. They used an autoregressive correlation structure in the GEE model, with the outcome measures as dependent variables. Time, group, and their interaction term (group × time) were incorporated into the model. A *P*-value <0.05 was considered statistically significant.

## RESULTS

### Demographic characteristics

We included 124 patients with cognitive dysfunction in brain injury, with 62 patients each in the intervention and control groups. During the study, seven patients withdrew: three from the intervention group (one due to hospital transfer following clinical deterioration, and two lost to follow-up because their hospitalisation period did not reach four weeks) and four from the control group (all discharged prematurely before completing the four-week intervention). The overall sample attrition rate was 5.6%. We handled missing data by multiple imputation, and comparisons between results after imputation and those from direct deletion of missing data showed no statistical differences, so we ultimately deleted the missing data (File S3 in the **Online Supplementary Document**). Finally, 117 patients completed the study (all receiving the full four-week intervention), comprising 59 patients in the intervention group and 58 patients in the control group (**Table 1**). Comparative analysis of baseline data revealed no significant differences between groups (all *P* > 0.05), confirming their comparability.

**Table 1:** Baseline characteristics of the study groups

	Intervention group, n (%)	Control group, n (%)	Statistics*	*P*-value
**Total number of participants**	59	58		
**Gender**				
Male	39 (66.1)	45 (77.6)	1.905	0.168
Female	20 (33.9)	13 (22.4)		
**Age, MD (IQR)**	72 (64, 77)	69 (59, 73)	−1.675†	0.094
**Marital status**				
Married	56 (94.9)	53 (91.4)	0.574	0.449
Other (unmarried/divorced/widowed)	3 (5.1)	5 (8.6)		
**Education**				
Illiteracy	4 (6.8)	2 (3.4)	4.577	0.333
Primary school	15 (25.4)	14 (24.1)		
Middle school	30 (50.8)	23 (39.7)		
High school	9 (15.3)	16 (27.6)		
College or above	1 (1.7)	3 (5.2)		
**Occupation (including before retirement)**				
Work of the state or institutions	29 (49.2)	23 (39.7)	1.771	0.413
Self-employed individual	9 (15.3)	14 (34.1)		
Farmer	21 (35.6)	21 (36.2)		
**Payment method**				
Medical insurance	59 (100.0)	58 (100.0)		
Self-pay	0 (0)	0 (0)		
**Family monthly income (in RMB)**				
<3000	2 (3.4)	1 (1.7)	0.893	0.640
3000–5000	11 (18.6)	8 (13.8)		
>5000	46 (78.0)	49 (84.5)		
**Operation**				
Yes	26 (44.1)	25 (43.1)	0.011	0.916
No	33 (56.9)	33 (56.9)		
**End of acute treatment (month)**				
<1	6 (10.2)	12 (20.7)	4.990	0.082
≥1 and <3	37 (62.7)	25 (43.1)		
≥3 and <6	16 (27.1)	21 (36.2)		
**Gender (caregiver)**				
Male	19 (32.2)	15 (25.9)	0.571	0.450
Female	40 (67.8)	43 (74.1)		
**Age (caregiver), x̄ (SD)**	55.4 (10.9)	54.2 (9.7)	0.818‡	0.368
**Relationship with patients**				
Spouse	27 (45.8)	28 (48.3)	4.468	0.215
Children	27 (45.8)	19 (32.8)		
Relatives and friends	4 (6.8)	6 (10.3)		
Caregiver	1 (1.7)	5 (8.6)		
**Education (caregiver)**				
Illiteracy	1 (1.7)	6 (10.3)	5.435	0.245
Primary school	7 (11.9)	6 (10.3)		
Middle school	34 (57.6)	25 (43.1)		
High school	9 (15.3)	11 (19.0)		
College or above	8 (13.6)	10 (17.2)		

IQR – interquartile range, MD – median, SD – standard deviation, x̄ – mean

*All statistics were from χ^2^ tests, unless specified otherwise.

†Mann-Whitney U test statistics.

‡Independent samples *t* test statistics.

### Comparison of primary and secondary outcomes between the two groups of patients before and after intervention

**Figure 2:**
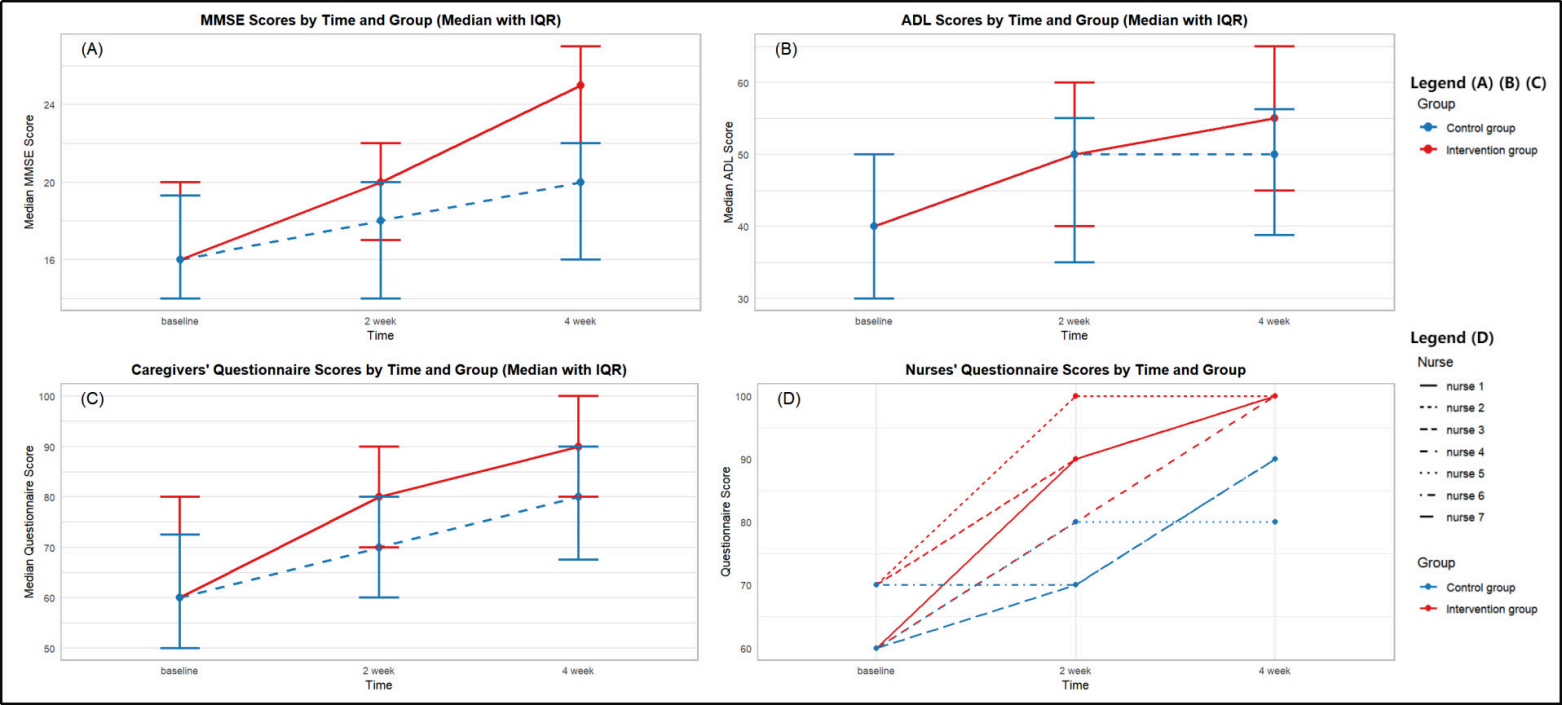
Mean changes in primary and secondary outcomes across T0–T2. **Panel A.** MMSE Scores by Time and Group (Median with IQR). **Panel B.** ADL Scores by Time and Group (Median with IQR). **Panel C.** Caregiver’s Questionnaire Scores by Time and Group (Median with IQR). **Panel D.** Nurses’ Questionnaire Scores by Time and Group. ADL – activities of daily living, IQR – interquartile range, MMSE – Mini-Mental State Examination, T0 – baseline, T1 – two-week intervention, T2 – four-week follow-up.

At both T1 and T2, MMSE scores increased in both groups, and the increase in scores of the intervention group was significantly higher than that of the control group (**Figure 2**, Panel A). The GEE results showed significantly higher cognitive function improvements in intervention group than control group at both T1 (b = 1.71; 95% CI = 0.76, 2.66; *P* < 0.001) and T2 (b = 2.71; 95% CI = 1.42, 4.00; *P* < 0.001), indicating that the intervention group maintained significantly better cognitive performance than the control group (**Table 2**).

**Table 2:** Changes in primary and secondary outcomes at T1 and T2 in the control and intervention group compared to T0 based on GEE models

	b	SE	95% CI	Wald χ^2^	*P*-value
**MMSE**					
Group	0.51	0.78	−1.01, 2.03	0.430	0.512
Time 1	1.29	0.30	0.71, 1.88	18.625	<0.001
Time 2	3.16	0.47	2.24, 4.07	45.526	<0.001
Group × Time 1	1.71	0.49	0.76, 2.66	12.360	<0.001
Group × Time 2	2.71	0.66	1.42, 4.00	17.010	<0.001
**ADL**					
Group	0.22	2.62	−4.92, 5.36	0.007	0.933
Time 1	4.14	0.81	2.55, 5.73	26.100	<0.001
Time 2	7.67	1.05	5.61, 9.73	53.365	<0.001
Group × Time 1	4.93	1.42	2.14, 7.72	11.988	0.001
Group × Time 2	5.46	1.69	2.15, 8.77	10.458	0.001
**Caregivers’ cognitive scores**					
Group	−0.20	4.24	−8.51, 8.11	0.002	0.962
Time 1	7.50	1.55	4.47, 10.53	23.470	<0.001
Time 2	12.76	2.30	8.26, 17.26	30.836	<0.001
Group × Time 1	8.09	2.71	2.78, 13.41	8.907	0.003
Group × Time 2	13.51	3.45	6.76, 20.27	15.369	<0.001

ADL – activities of daily living, CIs – confidence interval, GEE – generalised estimating equation, MMSE – Mini-Mental State Examination, SE – standard error, T0 – baseline, T1 – two-week intervention, T2 – four-week follow-up, χ^2^ – Chi-square

Results also showed a significantly greater increase in ADL (b = 4.93; 95% CI = 2.14, 7.72; *P* = 0.001) and caregivers’ cognitive scores (b = 8.09; 95% CI = 2.78, 13.41; *P* = 0.003) at T1, with sustained increases at T2 in ADL (b = 5.46; 95% CI = 2.15, 8.77; *P* = 0.001) and caregivers’ cognitive scores (b = 13.51; 95% CI = 6.76, 20.27; *P* < 0.001) (**Figure 2****,** Panels B and C).

Regarding nurses' cognitive scores, due to the small sample size (four nurses in the intervention group and three in the control group), we reported descriptive analysis. Both groups demonstrated improved cognitive knowledge scores following the intervention implementation, suggesting that clinical interventions helped enhance nurses' rehabilitation knowledge. At the two-week post-intervention assessment, all four nurses in the intervention group achieved scores of ≥80; only one nurse (33.3%) in the control group reached a score of ≥80 (**Figure 2**, Panel D; **Table 3**). At the four-week post-intervention assessment, all four nurses in the intervention group achieved perfect scores, and none of the three nurses in the control group attained perfect scores.

**Table 3:** Descriptive analysis of nurses' questionnaire scores

	Score (0 − 100)		
	**T0**	**T1**	**T2**
**Intervention group**			
Nurse 1	60	90	100
Nurse 2	70	100	100
Nurse 3	70	90	100
Nurse 4	60	80	100
**Control group**			
Nurse 5	60	80	80
Nurse 6	70	70	90
Nurse 7	60	70	90

T0 – baseline, T1 – two-week intervention, T2 – four-week follow-up

## DISCUSSION

Cognitive dysfunction represents the most prevalent sequela following brain injury [6]. Nevertheless, early rehabilitation efforts often prioritise physical impairments over cognitive recovery due to either subtle symptom presentation or clinical oversight of cognitive symptoms [28]. This delayed intervention significantly compromises treatment efficacy. Our results add to the strong evidence that early intervention for cognitive impairment should be further emphasised and strengthened.

Our results demonstrated that both the control and the intervention group of brain injury patients showed gradual improvement in MMSE scores during hospitalisation and rehabilitation. However, the intervention group exhibited a more pronounced improvement trajectory over time, suggesting that implementing evidence-based nursing protocols for early cognitive rehabilitation can effectively enhance cognitive recovery in brain injury patients, aligning with previous studies [29,30]. With this study, we provide nurses with systematic guidelines encompassing three key components: cognitive assessment, evidence-based interventions, and health promotion strategies. The superior cognitive improvement in the intervention group may be attributed to the timeliness and specificity of evidence-based interventions. Early cognitive training (*e.g.* memory drills and attention exercises) in the programme may stimulate neuroplasticity – the brain’s ability to reorganise neural pathways after injury [31]. By repeatedly activating relevant brain regions (*e.g.* the prefrontal cortex for executive function and the hippocampus for memory), the interventions may promote the formation of new synapses and compensate for damaged neural networks [32].

The ability to perform ADL represents the fundamental behavioural tasks required for individuals to maintain independent survival and adapt to their environment [33]. Our results show that the intervention group demonstrated superior ADL performance compared to controls. Through GEE analysis, a significant interaction effect emerged between ADL scores and time, indicating divergent recovery trajectories – the intervention group exhibited a steeper improvement gradient in ADL capacity throughout the rehabilitation period. These findings corroborate Brett and colleagues’ research documenting that brain injury patients with cognitive impairment frequently experience substantial ADL decline, often requiring external assistance for basic needs [34]. This functional deterioration compromises patients' quality of life, limits social participation, and imposes significant caregiving and socioeconomic burdens. As demonstrated in Xu and colleagues’ study [35], there is a strong correlation between cognitive function scores and ADL capacity in brain injury patients, with greater cognitive impairment predicting poorer functional independence. Moreover, the programme’s focus on ‘real-world’ skill training (*e.g.* dressing and undressing) bridges the gap between cognitive gains and practical application, enabling patients to transfer learned abilities to daily life more effectively than passive rehabilitation approaches. Thus, standardised early cognitive rehabilitation not only enhances ADL performance but also improves overall prognosis in this population.

The level of knowledge is one of the essential factors affecting the translation of evidence [36]. To provide correct nursing interventions, nurses need to acquire knowledge and skills in various aspects, such as clinical characteristics of the disease, types of complications, assessment, and intervention points. As primary implementers of rehabilitation care and the closest clinical partners for brain injury patients, nurses' enhanced cognitive care knowledge may critically improve patient outcomes [37]. Our results showed that the clinical application of the evidence-based nursing programme significantly increased the level of clinical nurses' knowledge about cognitive function rehabilitation. However, this finding should be interpreted with caution due to the small sample size of nurses involved in the assessment, which limits statistical power and generalisability. Additionally, as these nurses were directly involved in implementing the intervention, there may be potential performance bias or reporting bias that could influence the results. Further studies with larger, independent samples of nurses are needed to validate these preliminary observations.

In addition, the results also showed that the intervention had a significant effect on improving the knowledge scores of the patient's family members, which was much better than that of the control group. This may be because the study programme empowered patients' families through repeated health education, helping them understand the importance of cognitive rehabilitation and enabling them, as primary facilitators in wards, to support patients by guiding correct and standardised cognitive intervention methods [38]. Patients with cognitive dysfunction often struggle with attention, memory, and other limitations, making spontaneous, high-quality rehabilitation training difficult, while close family members – spouses or direct relations – can provide more targeted and supportive nursing interventions [39]. In the future, we will expand health education using information technology – such as ‘Internet+’, cloud computing, and virtual reality technology – to provide discharged patients and their families with more specialised and safe continuity of care services [40].

In summary, we implemented a best-evidence translational programme for brain injury rehabilitation. Results confirm the clinical value of evidence translation research and demonstrate the programme’s effectiveness in improving cognitive outcomes. However, evidence-based practice evolves dynamically and requires coordinating multiple factors, including best-available evidence, clinical context, provider judgment, and patient-family preferences [41]. Sustaining evidence application thus demands a dynamic assessment of influencing factors with timely refinements. Future work should include regular clinical reviews and large-scale studies to analyse programme sustainability, identify barriers and facilitators, and proactively address emerging challenges.

### Limitations

Several limitations should be acknowledged. First, due to practical constraints, we conducted a class of experimental studies that were unable to fully randomise patients to the intervention and control groups, leaving the results of the study open to potential confounding variables. However, we controlled for patient enrolment criteria and screening processes, so participants in the control and intervention groups did not appear to differ at baseline, which increased the internal validity of the findings. Second, while the cognitive rehabilitation knowledge questionnaire demonstrated robust content validity, its reliability remains unestablished due to resource constraints. Future studies should prioritise comprehensive psychometric validation, including test-retest reliability and internal consistency assessments, to strengthen measurement precision. Third, due to the time and effort constraints of the investigators, we did not conduct a multicentre experimental study design to further validate the study results, which reduced its external generalisability. Therefore, while the results demonstrate feasibility and preliminary efficacy, these findings must be interpreted with caution. Future multicentre trials with robust designs are needed before clinical implementation and dissemination.

## CONCLUSIONS

The evidence-based nursing interventions programme was effective in improving cognitive function and shows potential for clinical implementation and generalisation. A longer follow-up study is needed to evaluate its long-term effects on clinical outcomes.

## Additional Material


Online Supplementary Document
