# Supplementary material for: Evidence-based nursing interventions in cognitive dysfunction among adults with brain injury: a quasi-experiment
Source: J Glob Health. 2025 Aug 29;15:04253. doi: 10.7189/jogh.15.04253 (PMC12395861; doi:10.7189/jogh.15.04253)
Supplement: Online Supplementary Document [file jogh-15-04253-s001.pdf]

Supplement to: Gao Y, Zhou W, Wang Y, Zhou L. Evidence-based nursing interventions in cognitive dysfunction among adults with brain injury: a quasi-experiment. J Glob Health. 2025;15:04253.

Supplement1: Supplementary materials to “Evidence-based nursing interventions in cognitive dysfunction among adults with brain injury：A quasi-experiment”

Table of contents

Table S1. Evidence-based Nursing Intervention Program 2

Table S2. Summary of General Information on Experts 7

Table S3. Expert Authority Factor Evaluation 8

File S1. Brain Injury Cognitive Rehabilitation Nursing Knowledge Questionnaire (Nurses' Version) 9

File S2. Brain Injury Cognitive Rehabilitation Knowledge Questionnaire (Patient's Family Version) 12

File S3. Sensitivity Analysis of Missing Data 14

Checklist S1. SQUIRE 2.0 Statement—Checklist of items that should be included in reports of quality improvement studies 17

Table S1. Evidence-based Nursing Intervention Program

Item		Specifics	Frequency
**Assessment**	**Caveat**	1. Prior to cognitive assessment, assess the patient's state of consciousness; if the GCS score is >8, further assess cognitive function; if the GCS score is ≤8, then the patient is not suitable for cognitive screening, and monitor the patient's condition on a daily basis thereafter to avoid delaying the referral of the patient for a full cognitive assessment.<br>2. Conduct the first cognitive assessment within 24 hours of admission, taking into account as many factors as possible that contribute to cognitive deficits and limitations, including personal factors (e.g., age, education, etc.), preexisting medical conditions, and injury-related factors.	3 sessions (session 1: admission assessment; session 2: 2 weeks after intervention; session 3: 4 weeks after intervention)
	**Tools**	1. State of consciousness: Glasgow Coma Scale (GCS score)<br>2. Activities of Daily Living: ADL score (Activities of Daily Living)<br>3. Cognitive ability: Mini-Mental State Examination Scale(MMSE)<br>4. Nurse knowledge change: Brain Injury Cognitive Rehabilitation Nursing Knowledge Questionnaire<br>5. Family knowledge change: Brain Injury Cognitive Rehabilitation Knowledge Questionnaire	
**Interventions**	**Nurse role definition**	1. Patients and caregivers were instructed by nurses to perform and supervise appropriate early rehabilitation nursing interventions for cognitive function. The main performers of cognitive exercise in the ward are patients and caregivers, and nurses mainly assume the roles of instructors, health educators, and supervisors	
	**Formation of**	1. Form a multidisciplinary rehabilitation team (including neurosurgeons, physicians, rehabilitation	3 sessions (session 1:

**multidisciplinary teams**	physicians, physical therapists, occupational therapists, speech therapists, rehabilitation nurses, patients and caregivers)<br>2. Each member should have a clear division of labor, communicate and collaborate with each other, and hold regular team meetings to assess and set rehabilitation goals.<br>3. he multidisciplinary team will hold a total of 3 assessment meetings before intervention, after 2 weeks of intervention and after 4 weeks of intervention<br>4. Communicate in a timely manner about any changes or abnormalities in the patient's condition, and contact the speech therapist if speech disorders are found.	admission assessment; session 2: 2 weeks after intervention; session 3: 4 weeks after intervention)
**Cognitive functioning interventions**	Form specific, implementable interventions based on best evidence content, including:<br>1. with the participation of family members, cognitively reinforcing the patient's recognition of space, time, seasons, objects, and the environment through multiple repetitions of instruction and repetitions of instruction<br>2. guiding the patient to recall past events; after the patient's understanding of the basic information about time, space and objects is correct, the patient shall begin to train the patient's ability to perceive the similarities and differences of things, the limits of scope, the affinity of interpersonal relationships, as well as the judgment and extension of speech and behavior, etc., which is still carried out by means of repeated stimulation for many times<br>3. Patients should receive training on activities of daily living (ADL), and health education videos on position change, dressing and undressing, bed-wheelchair transfer, eating, grooming, walking, and walking up and down stairs should be pushed to patients through the TV in the wards (or	Planned frequency: at least 3 interventions per week for a total of 4 weeks Actual frequency: number of interventions per day X number of days of hospitalization

WeChat group); patients should first watch the videos repeatedly on their own after the videos have been distributed, and then the nurse-in-charge should explain, demonstrate, and provide guidance for the content of the health education and the content of the ADL training, respectively.

4. When strengthening cognitive training, information stimulation strategy (teaching patients intervention content with the help of visual images, verbal stimulation), metacognitive strategy (setting up clear rehabilitation goals, and carrying out cognitive interventions according to the form of planning-implementing-checking-reviewing), and distributed practice (the method of breaking down the task into smaller components; e.g., a) when training patients to wear clothes, they are first trained to button and put on the sleeves until patients can independently put on a piece of clothing; b when training patients in conversational communication, start with simple pronunciation, consciously train with patients in recognizing expressions of words and phrases and simple conversations, and gradually increase the level of difficulty according to the results of the training; c when training patients in reading, read simple words and phrases, then move to sentences, simple short texts, and finally texts that need to be comprehended)

5. provide patients with compensatory interventions, using environmental support (setting memos and reminders using tools such as cell phones, laptops, whiteboards, etc.) to strengthen patients' memories

6. during the intervention process, familiar and favorite songs, film and television episodes can be played for the patient, and family members can be instructed to contact the patient's limbs at irregular intervals to soothe the patient's emotions and facilitate cognitive function interventions;

		according to the patient's interest can be combined with structured music interventions, such as allowing the patient to drum rhythms with the rhythm of the music and so on<br>7. Physical exercise, combined with the patient's actual condition, in the patient's physical condition permits, recommended aerobic training (such as running, treadmill) or aerobic and resistance training, tai chi, qigong and other forms of exercise; if the patient's physical condition is poor, family members can be used to assist the passive exercise, such as passive joint exercises, from top to bottom, from near to far, left and right sides of the order of the upper limbs, lower limbs, each joint of the passive movement, supplemented by squeezing and compression, and the patient can be used to exercise. Passive exercise, supplemented by squeezing and weight training, repeated passive exercise for each joint less than 10 of normal mobility, avoid keeping it in the same position for a long time, and strengthen the passive activities of the limbs according to certain time intervals; the frequency and intensity of the exercise program (including active exercise and auxiliary passive exercise) should be determined by the doctor's instructions, and ideally it should be continued for more than four weeks, and the patient should exercise at least three times a week for 30 minutes each time.	
**Health education**	**Health Education Evaluation Team**	1. A health education evaluation team was set up, with two senior nurses responsible for the evaluation of patients' health education, and a weekly shift was arranged to check whether the health education of all patients was in place.<br>2. Evaluation content includes: admission/migration education, disease education, dietary education, drug management, exercise intervention, plumbing care, skin care, awareness of disease	3 sessions (session 1: admission assessment; session 2: 2 weeks after intervention; session 3: 4 weeks

	characteristics and importance of education, specific cognitive intervention content and method of education in the program.	after intervention)
**Approach**	Responsible nurses follow the content of health education in order to explain; the process of education is tailored to the individual, for patients and caregivers with low literacy and acceptance, the education time is appropriately extended every day, and bedside explanations, public pushes (related to cognitive function training), and teaching demonstrations are adopted as educational approaches	
**Pre-discharge education**	1 day before discharge, ask the patient or caregiver to instruct the patient to perform cognitive function exercise once, and give proper evaluation and advice on the exercise method, and inform the follow-up requirements	

Table S2. Summary of General Information on Experts

No.	Gender	Age	Education	Title	Specialized field	Years of work	Trained in rehabilitation
1	Female	56	master's degree	Chief Nurse	Evidence-Based Nursing/ Nursing Management	35	Yes
2	Female	53	doctor's degree	Professor	Evidence-Based Nursing/ Clinical Nursing	32	No
3	Female	39	master's degree	Nurse Practitioner-in-Charge	Rehabilitation Nursing	13	Yes
4	Female	41	bachelor's degree	Nurse Practitioner-in-Charge	Rehabilitation Nursing	20	Yes
5	Male	56	doctor's degree	Chief Physician	Clinical Medicine	30	Yes
6	Male	48	master's degree	Attending Physician	Clinical Medicine	23	No
7	Male	42	doctor's degree	Attending Physician	Clinical Medicine	15	Yes
8	Female	47	master's degree	Chief Rehabilitator	Clinical Rehabilitation	20	Yes
9	Female	42	bachelor's degree	Head Rehabilitator	Clinical Rehabilitation	20	Yes
10	Male	39	master's degree	Head Rehabilitator	Clinical Rehabilitation	14	Yes

Note:A total of 10 experts; 4 with senior titles and 6 with intermediate titles; average age 46, average years of experience 22 years; 4 experts in the field of nursing, 3 experts in the field of clinical medicine, 3 experts in the field of rehabilitation therapy

Table S3. Expert Authority Factor Evaluation

No.	expert judgment				Expert judgment Coefficient(Ca)	Expert familiarity Coefficient(Cs)	Degree of expert authority Coefficient(Cr)
	A1	A2	A3	A4			
1	0.5	0.3	0.1	0.1	1	1	1
2	0.5	0.3	0.1	0.1	1	1	1
3	0.4	0.3	0.1	0.1	0.9	0.8	0.85
4	0.5	0.2	0.1	0.1	0.9	0.8	0.85
5	0.5	0.3	0.1	0.1	1	1	1
6	0.5	0.3	0.1	0.1	1	0.8	0.9
7	0.4	0.3	0.1	0.1	0.9	1	0.95
8	0.5	0.3	0.1	0.1	1	0.8	0.9
9	0.5	0.1	0.1	0.1	0.8	0.8	0.8
10	0.5	0.2	0.1	0.1	0.9	0.8	0.85
总计	0.48	0.26	0.1	0.1	0.94	0.88	0.91

Note: A1: Practical and work experience; A2: Theoretical analysis; A3: Domestic and international literature/peer understanding; A4: Subjective intuitive judgment

File S1. Brain Injury Cognitive Rehabilitation Nursing Knowledge Questionnaire (Nurses' Version)

Dear Nurse:

Greetings! In order to enhance the efficacy of brain injury rehabilitation nursing care and improve the health outcomes and quality of life of patients, a research team headed by Prof. Lanshu Zhou at the School of Nursing, Naval Medical University, is conducting a study on brain injury rehabilitation nursing care. This study is the Army Nursing Innovation and Cultivation Special Program Innovation Project (2021HL021). The study aims to apply the best evidence of rehabilitation nursing care for brain injuries to clinical practice through evidence-based research and expert meetings, implement rehabilitation nursing protocols to standardize nursing practice to improve rehabilitation outcomes, promote the enhancement of nurses' related knowledge and skills, and provide clinical managers with a professional basis for decision-making.

We invite you to participate in this questionnaire survey, which is a survey on the knowledge of rehabilitation nursing care for brain injuries, and this study aims to explore the influencing factors and interventions of current rehabilitation nursing care for clinical patients, and we hope to have your assistance. Whether or not you participate in this study is entirely up to you, and your participation or non-participation will not have any adverse effects on you. The study is anonymous, your personal data will be kept strictly confidential and the findings will be used for report writing. It will take you approximately 15 minutes to complete this questionnaire. Please read the items in the questionnaire below carefully and put a tick in the appropriate place, please do not miss any item. Thank you for your cooperation!

(I) General Information

1. Gender: ①Male ②Female

2.Age:

3. Hospital where:

4. Education: ① secondary school or college ② undergraduate ③ graduate and above

5. Years of working experience in neurosurgery:

6. Title: ①Nurse ②Nurse Practitioner ③Supervisory Nurse and above ⑤Physician ⑥Attending Physician and above ⑦Rehabilitation Therapist

7. Position: ① Director of Nursing Department ② Head Nurse ③ Teaching Nurse ④ Nurse ⑤ Doctor ⑥ Rehabilitation Therapist ⑦ Other-

8. Whether they have received relevant rehabilitation training: ① Yes ② No

(II) Survey on nursing knowledge of cognitive function rehabilitation for brain injury

1. When should patients with brain injury be assessed for cognitive function after hospitalization:

A. Within 4 hours B. Within 6 hours C. Within 8 hours D. Within 24 hours E. Don't know

2.When can rehabilitation care for cognitive function be initiated:

A. 24h after admission

B. 24h after stabilization of neurological function

C. 48h after admission to hospital

D. 24h after admission to ICU

E. Don't know

3. Cognitive function assessment tools for patients with craniocerebral injury do not include:

A. MMSE scale B. TCA cognitive assessment C. MOCA scale D. CCSE cognitive screening E. Ashworth scale

4.Which of the following instruments is closely related to the patient's literacy level:

A. MMSE scale B. MOCA scale C. ADL scale D. Picture-in-Picture test E. Don't know

5.What is true about GCS score of Glasgow Coma Scale is as follows:

A.13 to 15 is classified as severe impairment

B.12 to 15 is classified as mild impairment

C.9 to 12 is classified as moderate impairment

D.3 to 8 is classified as mild impairment

E. Don't know

6. Cognitive therapy does not include:

A. recognizing automatic thoughts B. recognizing cognitive errors C. reality testing

D. getting rid of attention E. cognitive training

7.A routine test to perform diagnosis and differentiation of cognitive function is:

A. CT B. single photon emission computed tomography C. positron emission computed tomography D. magnetic resonance imaging E. don't know

8. Common methods of attention training do not include:

A. guessing games B. music therapy C. time sense training D. deletion of homework E. don't know

9.Clinical treatments used to improve cognitive function do not include:

A. physical therapy B. neuropsychotherapy C. speech therapy

D. occupational therapy E.Don't know

10. The following statement about cognitive function rehabilitation nursing is correct:

A. appropriate cognitive function training is selected according to the different conditions of patients

B. there is no need to assess the patient's awareness and level of cooperation before cognitive function rehabilitation care

C. decontextualized therapy should be emphasized to maximize the restoration of patient function and self-awareness

D. dual-task training is advocated to maximize patient functioning and self-awareness in grayscale

E. don't know

File S2. Brain Injury Cognitive Rehabilitation Knowledge Questionnaire (Patient's Family Version)

Dear patient's family:

Greetings! We aim to understand your knowledge about cognitive dysfunction after craniocerebral injury, in order to improve the quality of our services and provide better care for patients. Please fill in the questionnaire according to your actual knowledge.

Please fill in the questionnaire according to your actual knowledge, thank you for your participation!

(I) General Information

1. Gender: ① male ② female

2. Age

3. Education: ① junior high school and below ② junior college or college ③ bachelor's degree ④ graduate school and above

4. Relationship with patients:

(II) Knowledge survey

**Make a judgment on the following questions by checking the judgment box (√)**	**Right**	**wrong**	**I don't know**
1. Patients with cognitive impairment may have problems with memory, learning, judgment, and communication.			
2. patients with brain injury are prone to cognitive impairment.			
3. Cognitive impairment is not a major problem and does not require special rehabilitation work.			
4. Patients with both physical and cognitive problems should first solve their physical problems before considering cognitive rehabilitation.			
5. Cognitive rehabilitation should be carried out as early as possible.			
6. Cognitive rehabilitation is the responsibility of doctors and nurses, and the family cannot do anything about it.			

7. Cognitive impairment does not affect the patient's basic life.			
8. Family members are important in the cognitive rehabilitation of the patient and will contribute to the patient's recovery.			
9. Cognitive problems are the main reason for the patient's later problems such as wandering, loss of visits, and injuries.			
10. After the patient is discharged from the hospital, family members should continue to help the patient with cognitive rehabilitation.			

File S3. Sensitivity Analysis of Missing Data

In this study, 124 patients with cognitive dysfunction in brain injury were screened from the database at the beginning, of which 7 cases were lost in follow-up and had missing data for dependent variables, so only the 7 cases were interpolated using multiple interpolation in this study.

We used listwise deletion for the 5.6% of missing observations in the body submission. Meanwhile, we have conducted a sensitivity analysis using multiple imputation (MI) to address potential bias due to missing data. Specifically, we performed multiple imputation with m = 5 imputed datasets using the mice package in R, incorporating all relevant baseline covariates into the imputation model. Subsequently, we fitted the GEE models using both the original dataset (with complete cases only) and the pooled results from the multiply imputed datasets.

The comparison between the two approaches showed no substantial differences in parameter estimates or statistical significance, indicating that the conclusions are robust to the method of handling missing data. This supports the validity of our original findings and suggests that the impact of missing data was minimal in our study. There were no substantial changes in the results, indicating that the results of this study are stable (see eTable 1)

eTable 1. Comparison of generalized estimating equations model for deleting missing data and interpolating missing data

Outcomes	Model 1(deleting missing data)						Model 2(multiple imputation to process incomplete data)					
	B	SE	95% CI		Wald χ^2	P	B	SE	95% CI		Wald χ^2	P
			lower	upper					lower	upper		
**MMSE**												
Group	0.51	0.78	-1.01	2.03	0.430	0.512	0.51	0.76	-0.98	2.01	0.450	0.502
Time 1	1.29	0.30	0.71	1.88	18.625	<0.001	1.38	0.29	0.81	1.94	22.606	<0.001
Time 2	3.16	0.47	2.24	4.07	45.526	<0.001	3.26	0.45	2.37	4.15	51.910	<0.001
Group*time 1	1.71	0.49	0.76	2.66	12.360	<0.001	1.65	0.46	0.75	2.55	12.780	<0.001
Group*time 2	2.71	0.66	1.42	4.00	17.010	<0.001	2.57	0.63	1.34	3.80	16.801	<0.001
**ADL**												
Group	0.22	2.62	-4.92	5.36	0.007	0.933	-0.25	2.67	-5.49	4.98	0.009	0.924
Time 1	4.14	0.81	2.55	5.73	26.100	<0.001	4.28	0.78	2.75	5.80	30.210	<0.001
Time 2	7.67	1.05	5.61	9.73	53.365	<0.001	7.86	1.02	5.85	9.86	58.845	<0.001
Group*time 1	4.93	1.42	2.14	7.72	11.988	0.001	4.26	1.37	1.56	6.95	9.59	0.002
Group*time 2	5.46	1.69	2.15	8.77	10.458	0.001	4.98	1.62	1.81	8.16	9.45	0.002
**Caregivers' cognitive scores**												

Group	-0.20	4.24	-8.51	8.11	0.002	0.962	-0.21	4.07	-8.19	7.77	0.003	0.959
Time 1	7.50	1.55	4.47	10.53	23.470	<0.001	7.15	1.51	4.19	10.11	22.394	<0.001
Time 2	12.76	2.30	8.26	17.26	30.836	<0.001	12.31	2.22	7.96	16.66	30.801	<0.001
Group*time 1	8.09	2.71	2.78	13.41	8.907	0.003	8.61	2.60	3.51	13.71	10.947	0.001
Group*time 2	13.51	3.45	6.76	20.27	15.369	<0.001	13.32	3.37	6.71	19.93	15.60	<0.001

Checklist S1. SQUIRE 2.0 Statement—Checklist of items that should be included in reports of quality improvement studies

Checklist Item	Checklist Item	Page
1. Title	Indicate that the article concerns an initiative to improve health care (broadly defined to include the quality, safety, effectiveness, patient-centeredness, timeliness, cost, efficiency, and equity of health care)	Page 1 (Title)
2. Abstract	a. Provide adequate information to aid in searching and indexing	Page 1-2 (Abstract)
	b. Summarize all key information from various sections of the text using the abstract format of the intended publication or a structured summary as background, local problem, methods, interventions, results, conclusions	
3. Problem description	Nature and significance of the local problem	Page 1-2 (Abstract)
4. Available knowledge	Summary of what is currently known about the problem, including relevant previous studies	Page 3 (Introduction)
5. Rationale	Informal or formal frameworks, models, concepts, and/or theories used to explain the problem, any reasons or assumptions that were used to develop the intervention(s), and reasons why the intervention(s) was expected to work	Page 4 (Introduction)
6. Specific aims	Purpose of the project and of this report	Page 4 (Introduction)
7. Context	Contextual elements considered important at the outset of introducing the intervention(s)	Page 4 (Study design)
8. Intervention(s)	a. Description of the intervention(s) in sufficient detail that other(s) could reproduce it	Page 4 (Study Design\ Intervention Team Composition); Table S1
	b. Specifics of the team involved in the work	
9. Study of the intervention(s)	a. Approach chosen for assessing the impact of the intervention(s)	Page 5 (Study subjects)
	b. Approach used to establish whether the observed outcomes were attributable to the intervention(s)	
10. Measures	a. Measures chosen for studying processes and outcomes of the intervention(s), including rationale for choosing them, their operational definitions and their validity and reliability	Page 6-8 (Measurements)
	b. Description of the approach to the ongoing assessment of contextual elements that contributed to the success, failure, efficiency, and cost	
	c. Methods used for assessing completeness and accuracy of data	
11. Analysis	a. Qualitative and quantitative methods used to draw inferences from the data	Page 8 (Data analysis)
	b. Methods for understanding variation within the data, including the effects of time as a variable	
12. Ethical considerations	Ethical aspects of implementing and studying the intervention(s) and how they were addressed, including but not limited to formal ethics review and potential conflicts of interest	Page 14 (Ethics)
13. Results	a. Initial steps of the intervention(s) and their evolution over time (eg, time-line diagram, flow chart, or table), including modifications made to the intervention	Page 17-19 (Results); Page 20-23

	during the project	(Table 1-3)
	b. Details of the process measures and outcomes	
	c. Contextual elements that interacted with the intervention(s)	
	d. Observed associations between outcomes, interventions, and relevant contextual elements	
	e. Unintended consequences such as unexpected benefits, problems, failures, or costs associated with the intervention(s)	
	f. Details about missing data	
14. Summary	a. Key findings, including relevance to the rationale and specific aims	Page 10 (Discussion)
	b. Particular strengths of the project	
15. Interpretation	a. Nature of the association between the intervention(s) and the outcomes	Page 10-12 (Discussion)
	b. Comparison of results with findings from other publications	
	c. Impact of the project on people and systems	
	d. Reasons for any differences between observed and anticipated outcomes, including the influence of context	
	e. Costs and strategic trade-offs, including opportunity costs	
16. Limitations	a. Limits to the generalizability of the work	Page 13 (Limitations)
	b. Factors that might have limited internal validity such as confounding; bias; or imprecision in the design, methods, measurement, or analysis	
	c. Efforts made to minimize and adjust for limitations	
17. Conclusions	a. Usefulness of the work	Page 13 (Conclusions)
	b. Sustainability	
	c. Potential for spread to other contexts	
	d. Implications for practice and for further study in the field	
	e. Suggested next steps	
18. Funding	Sources of funding that supported this work; role, if any, of the funding organization in the design, implementation, interpretation, and reporting	Page 14 (Funding)
